# Evaluation of the Effects of Telepsychotherapy in the Treatment and Prevention of Eating Disorders in Adolescents

**DOI:** 10.3390/ijerph182312573

**Published:** 2021-11-29

**Authors:** Marilena Maglia, Graziana Corello, Pasquale Caponnetto

**Affiliations:** 1 Department of Educational Sciences, University of Catania, 95131 Catania, Italy; p.caponnetto@unict.it; 2 Center of Excellence for the Acceleration of Harm Reduction (COEHAR), University of Catania, 95123 Catania, Italy; 3 CTA-Villa Chiara Psychiatric Rehabilitation Clinic and Research, 95030 Mascalucia, Italy; grazianacorello@live.it

**Keywords:** telepsychotherapy, e-health, prevention of eating disorders, adolescents, anorexia nervosa, bulimia nervosa

## Abstract

According to the WHO definition, “telemedicine is the provision of health services, where distance is a critical factor, by all health professionals who use information and communication technologies for the exchange of valid information for the diagnosis, treatment and prevention of diseases, research and evaluation, and for the continuous training of health professionals, all in the interest of advancing the health of individuals and their communities”. The purpose of our review work is specifically to investigate the effects of telemedicine in the treatment and prevention of eating disorders in adolescents. From June 2021 to (September 2021) in the databases of the Web of Science, EMBASE, PsycINFO and CINHAL, using search terms such as telehealth, eating disorder, adolescents, Internet/online treatments CBT and FB-T, anorexia nervosa, bulimia nervosa and binge eating disorder. The articles resulting from the search phases in the databases listed above produced a total of 176 items. Once the procedures for selecting the works were completed, only four studies were included in the review. Modern e-health psychological approaches in the treatment of eating disorders provide potential bases of continuous assistance that are decidedly less burdensome in the costs of territorial services in the case that they are not identified as necessary.

## 1. Introduction

According to the WHO definition, “telemedicine is the provision of health services, where distance is a critical factor, by all health professionals who use information and communication technologies for the exchange of valid information for the diagnosis, treatment and prevention of diseases and injuries, research and evaluation, and for the continuous training of health professionals, all in the interest of advancing the health of individuals and their communities” [1], as it allows a constant and interactive communication in real time between patient and therapist, at any time, overcoming the physical distance.

For several years, the use of the Internet and telemedicine for the treatment of psychological disorders has spread, exploiting advances in technology to make prevention programs and therapeutic interventions more accessible and feasible.

These therapeutic interventions could improve the treatment and prevention of eating disorders, as they are generally easier to disseminate, cheaper and extended to individuals who otherwise would not have the opportunity to access specialized care [2].

Better access to care and stress reduction with the use of telemedicine can also increase patient satisfaction [3].

They may be appropriate for working with adolescents, adaptable to the individual needs of each patient and require less use of skilled personnel, thereby disposing of the workload of healthcare [4,5,6,7].

However, a variable that affects virtual therapeutic interventions is the extent and frequency with which the interaction between people takes place in certain conditions [8]; it is important that at least a short and regular therapeutic contact is provided, otherwise it is more likely that high rates of abandonment and a reduced effectiveness of treatment will occur [9].

Following the COVID-19 pandemic, telemedicine has become the standard therapy for the treatment of psychological disorders, including eating disorders (through video conferences, telephone support, e-mail, mobile apps, training on online platforms) [10], except in those more serious cases where direct hospital intervention was necessary.

Importantly, research carried out during the COVID-19 pandemic identifies telemedicine as a promising alternative for the provision of outpatient care [11].

In addition, an Australian study, aimed at detecting the effects of the transition from classic face-to-face therapy (CBT-ED or FBT, the latter adopted with adolescents) to sessions conducted through telepsych, showed that patients achieved great improvements in the reduction in primary symptoms related to the eating disorder and secondary symptoms (anxiety and depression), positively evaluating the quality of treatment and the therapeutic alliance [12].

The COVID-19 pandemic represents a global catastrophe with serious economic, political, social and above all, health consequences, causing the death of millions of people worldwide [13].

In addition to the morbidities and mortality of those infected, the pandemic has had a strongly negative impact on the mental health of the population, not only with the increase in psychic distress in people with pre-existing psychopathologies, but also in health workers and in the general population; in particular, increases in depression, anxiety and post-traumatic stress disorder (PTSD) symptoms have been detected [14,15].

The negative effects of the pandemic have also spread to people with eating disorders: 40% of individuals reported a worsening of symptoms after a couple of weeks of the lockdown, due in part to the interruption of the day hospital treatments they needed, and about 60% manifested anxiety symptoms in comorbidity [16]. In a survey conducted in Australia starting in April 2020, it was found that, in the group of participants with eating disorders, 35.5% reported an increase in binge eating, while 18.9% reported an increase in food elimination behaviors [17].

Adolescents represent an extremely vulnerable part of the population in the development of psychological disorders since they are more exposed to the negative effects of the pandemic, social isolation and imposed restrictions, at a stage of life in which the relationship and comparison with peers are crucial for the construction of their identity.

In addition, the confinement due to the pandemic has greatly increased the time spent by adolescents on social networks, showing that the latter are related to eating disorders in this age group [18,19,20].

The pandemic has also had negative consequences on the mental health of children and adolescents. Several studies found, the increase in anxiety and depressive symptoms, respectively 37.4% and 43.7%, in young people aged between 12 to 18 years, as well as in adults [21], and an incidence of somatic symptoms of 2.39% among Chinese primary school students [22].

Nutrition and eating disorders often arise precisely in the period of adolescence, spreading especially in Western societies [23], with the tendency to persist and worsen in adulthood, sometimes evolving into a psychiatric disorder if one does not intervene promptly before this phase of life.

A cohort study evaluated 48 adolescents between 9 and 17 years old during the pandemic period (April to October 2020), comparing them with the data that the care center recorded in the year before COVID-19, and in 40% (n = 19) of participants, the effects of the pandemic and the subsequent lockdown were identified as the direct triggers for the onset of patients’ pathology and arrival at the emergency room, with a greater impairment of physical health and a more acute onset of the disease, compared with those who did not indicate the effects of the pandemic among the causes of their eating disorder (duration of the disease 5.59 months vs. 11.63 months).

In addition, the significant impact of the pandemic on the increase in eating disorders in adolescents was measured by comparing the number of urgent hospitalizations during the same period with the previous year, which increased by 63% [24]. The increase in visits and hospitalization compared to the year before COVID-19 was detected in a study involving children and adolescents with eating disorders in order to evaluate the effectiveness of treatments combined with telepsychotherapy for outpatients of a day-hospital. Participants previously assisted by the day hospital required a significantly greater number of medical consultations (both telematics and in presence), with 41.9% of patients experiencing confinement, which showed a reactivation of the psychopathological symptoms associated with the disease, such as food restriction, excessive exercise, worries and fear of gaining weight. In addition, increased mood disorders, self-aggressive behaviors and suicide risk were the main causes of hospitalization [25].

There is much evidence in clinical practice attesting the benefits and improvements in health in patients with Bulimia Nervosa (BN) who are treated with Cognitive Behavioral Therapy (CBT) and family therapies [26,27,28]. A recent randomized clinical trial [29] conducted with adolescents with BN compared the effects of CBT-A adapted for adolescents [30], and a specific form of familial treatment with Family-Based Treatment Bulimia Nervosa (FBT-BN) [31], which showed that at the 12-month follow-up, there were no statistically significant differences in the abstinence rate of binge eating, while at the end of treatment and at the 6-month follow-up, there was a significantly higher abstinence rate of binge eating in the FBT-BN group.

Comparable effects between CBT and FT were also achieved for the treatment of Anorexia Nervosa (AN) in young adults in another randomized clinical trial [32], in which an increase in the BMI from baseline to end of treatment was detected in both treatment groups, showing that both treatments are highly effective in young adults with AN in terms of restoring weight-reducing eating disorders and improving psychological well-being.

To the best of our knowledge, the evidence for the acceptability and efficacy of e-health interventions in DIs is limited. Self-monitoring functions offer control and analysis of ED-related symptoms and thus help increase patients’ conscious involvement in treatment [33]. Among existing e-health interventions, Internet-based cognitive behavioral therapy and guided self-help are considered two of the most effective approaches in reducing the psychopathology named eating disorder, also considering the frequent interaction between individuals with ED in the online health communities [34,35].

There are multiple pieces of evidence in the literature of the actual benefits of telepsychotherapy applied to the various forms of CBT for the treatment of eating disorders, but the majority of these studies are based on adults [36,37,38] or include both adolescents and adults in the sample [39].

A recent RCT study [40], in which intervention in patients with ED was evaluated through a fully automated feedback and self-monitoring system, found statistically significant reductions in ED symptomatology but also in psychiatric comorbidities (anxiety, depression and persevering thinking) compared to the waitlist control group.

The insufficiency of studies conducted with adolescents does not allow the findings to be extended and generalized to include the positive effects of telepsychotherapy on these special populations, so future research should deepen these areas of intervention.

To the best of our knowledge, no reviews have been conducted specifically on effects of telepsychotherapy on the treatment and prevention of eating disorders in adolescents. Our aim was to focus specifically on effects of telepsychotherapy on the treatment and prevention of eating disorders in adolescents, providing an updated view of the current spectrum of available therapeutic techniques and their effectiveness in clinical practice.

## 2. Materials and Methods

### 2.1. Research Object

The aim of this research was to determine the effectiveness of therapeutic and prevention interventions for eating disorders in adolescents, as delivered through telepsychotherapy (Internet, mobile apps, video conferences, email).

### 2.2. Search Strategy

The review was carried out completely according to the PRISMA 2020 guidelines for systematic review by the PRISMA Group [41]. The bibliographic search was carried out from June 2021 until the date of submission of the article (September 2021). MM, PC and GC searched the databases Web of Science, EMBASE, PsycINFO and CINHAL for relevant studies using the following search terms string: (“telepsychotherapy” OR telepsychology Or telehealth) AND (eating disorder* OR anorexia OR bulimia* OR binge eating disorder* OR “adolescent” OR “Internet/online treatments CBT and FB-T”). The electronic searching was supplemented by the hand-searching of reference lists of the included review articles to identify any additional source.

### 2.3. Elegibility Criteria

We included articles written in English language meeting the following criteria:

1. Participants: adolescents diagnosed with eating disorders, as the main recipients of the interventions, and adolescents who met the risk criteria for anorexia, bulimia and binge eating disorder.
2. Intervention: included interventions using telepsychotherapy for the delivery of therapeutic treatments (CBT and FBT) through video conferencing, smartphone apps, online questionnaires for qualitative research, a web-based prevention program for parents of adolescents who met the risk criteria for anorexia, bulimia and binge eating disorderAN.
3. Comparison: therapy as usual (TAU), wait-list control condition.
4. Outcomes: body image satisfaction was considered for the results; increased body weight in an; reduction in psychopathological symptoms of the eating disorder (deliberate and planned weight control, food restriction due to weight concerns, excessive frequency of diets, vomiting, fasting, use of laxatives, tablets to lose weight, skipping meals) and secondary symptoms (anxiety, depression, self-esteem); reduction in anorexia, bulimia and binge eating disorderAN risk factors in prevention interventions.
5. Study design: clinical trials, randomized clinical trial, review, systematic review, books and document.

### 2.4. Data Extraction

The reviewers MM and PC extracted data using a format which included: author, year, title, country where the research took place, type of study, sample, measurements used, characteristics of studies, type of intervention, target symptoms to be treated, frequency and duration of the interventions, outcomes and their assessments, follow-up if present.

## 3. Results

**Figure 1:**
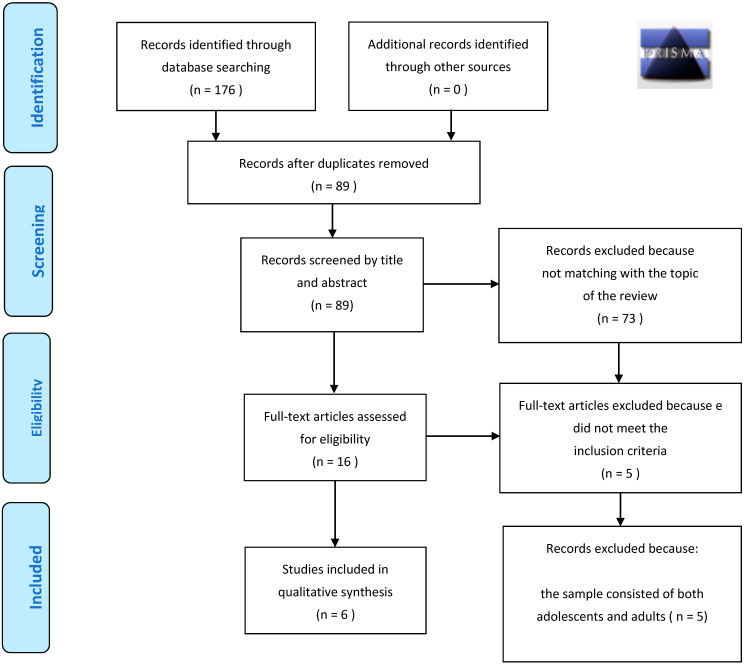
PRISMA 2009 Flow Diagram.

The articles resulting from the search phases in the databases listed above produced a total of 176 articles. After this first search, 87 duplicates were eliminated, thus identifying 89 articles. These articles were subjected to further analysis through the reading of the title and the abstract, which led to the elimination of another 73 articles that not compatible with the objectives of the review. The remaining 16 articles were examined in full, 5 of which were excluded because they were deemed not entirely relevant to the inclusion criteria and 5 were excluded because the sample consisted of adolescents and adults. Finally, 6 studies included in the review. The small number of studies, conducted in this field of research exclusively with adolescents, shows how necessary it is to carry out continuous research aimed at investigating the effects of telepsychotherapy and interventions that exploit new technologies in the study of eating disorders. The above description is summarized in the flowchart in Figure 1, while data extraction from these studies can be viewed in Table 1.

### 3.1. Schemes

#### 3.1.1. Heinicke et al. (2007)

The study by Heinecke et al. [42] represents one of the first RCT studies aimed at evaluating the effects of an online group program aimed at improving the satisfaction of body image and symptoms associated with eating disorders, compared with control group, with an established follow-up of 2 and 6 months from the end of the program.

The participants were 73 adolescent girls (mean age of 14.4 years) divided into the intervention group (N = 36) and control group with delayed treatment (N = 37). Inclusion criteria were: female; age range between 12 and 18 years; attending high school (classes 7–12); self-identification of eating problems or dissatisfaction with body image; access to the Internet.

Body dissatisfaction was assessed with the Body Shape Questionnaire Short Form (BSQ-F) [43] and the body comparison scale. The Dutch Eating Behavior Questionnaire Restraint subscale (DEBQ-R) [44] and the Extreme Weight Loss Behaviors (EWLB) scale [45] were used to assess eating disorders, while the EDI Bulimia (EDI-B) subscale [46] assessed bulimic symptoms. The short form of the Beck Depression Inventory (BDI-SF) was also administered [47] in order to assess depressive symptoms.

The intervention program [42] My Body, My Life: Body Image Program for Adolescent Girls, based on the principles of CBT, consisted of six weekly synchronous online sessions of 90 min, in a secure and password-protected chat-room, in small groups (4–8 people) and was facilitated by a qualified therapist and a guided self-help manual, composed of a psychoeducational part with activities corresponding to each session that the participants had to read and complete. A discussion forum served as a permanent bulletin board for participants who wished to communicate during the week, and at the same time, the therapist could leave messages for the group. The content of the sessions was focused on examining the motivation for change; the issue of body image; examining irrational beliefs and negative thoughts about the body; food experiences and unhealthy eating patterns; teaching self-monitoring techniques; investigating the correlation between depression, self-esteem, interpersonal relationships; and providing relapse prevention strategies. At the end of the program, questionnaires were sent to the intervention group at the 2- and 6-month follow-ups.

At the end of the program, significant improvements in measures of body dissatisfaction and symptoms of eating disorder (T2) were observed in the intervention group, with scores close to those of non-clinical samples. The tendency towards body comparison has also decreased significantly. These results were also maintained at the 2- and 6-months follow-ups. In addition to these positive results, depressive symptoms also decreased, probably also due to the assiduous interaction of the girls in the group, in which they probably found encouragement and peer support.

In the participants’ final qualitative assessment, 65% reported preferring to participate in an Internet-broadcast program, 15% would prefer a face-to-face program and 20% were undecided or considered them both enjoyable. In addition, 88% reported that the program contributed moderately or markedly to improve their eating patterns in a positive way, and only two people thought it had been useless.

Heinecke’s study also has these limitations, as it would have been useful to compare the results obtained with a control group, in which the program was carried out in face-to-face mode; without this control condition, it is not possible to establish for certain whether the observed improvements were due to non-specific treatment effects.

#### 3.1.2. Anastasiadou et al. (2020)

This multicenter randomized controlled trial [48] evaluated the clinical efficacy of a combined mHealth intervention for eating disorders (ED) with cognitive behavioral therapy (CBT).

A total of 106 patients were randomly assigned to two parallel groups. Patients in the experimental group (N = 53; mean age 17.25) received standard face-to-face CBT plus a mobile intervention through an application called “TCApp,” which provides self-monitoring and an online chat with the therapist, and the control group (N = 53) received only standard face-to-face CBT (therapy as usual, TAU). ED-related symptoms were assessed with the Eating Disorder Examination Questionnaire (EDE-Q) [49], and the Short Evaluation of Eating Disorders (SEED) [50], while Beck Depression Inventory (BDI-II) [51] and State-Trait Anxiety [52], EuroQoL-EQ-5L (EQ-5D-5L) [53], were administered to measure psychological comorbidities.

The TCApp used in the study was built specifically for people with ED, based on the principles of CBT: it connects therapist and patient 24 h a day, uses online food records, monitors thoughts, actions and emotions with continuous self-monitoring, and at the same time, therapists can view the progress of their patients by using graphs and reports and contact them in case of emergencies.

Spanish participants were recruited by the different mental health services, public and private, and all were diagnosed with an eating disorder according to DSM 5 diagnostic criteria.

The experimental group received a standard face-to-face CBT, in addition to using the TCApp mobile system daily for 12 weeks. The control group received standard treatment based on traditional face-to-face CBT for the same period of time.

The results [48] show that there was no significant difference between the two groups on longitudinal changes in total and subscale EDE-Q scores or in the severity index for AN and BN as assessed by SEED. CBT determined reductions in ED-associated symptoms in both groups.

However, a significant effect of the intervention on longitudinal changes was found in the variable “total number of regular visits” in the experimental group, i.e., the number of visits to the mental health unit was lower in treatment than compared with control group, suggesting that the use of telepsychotherapy can also lead to a reduction in healthcare costs associated with face-to-face visits.

Hence, the use of smartphone apps, such as TCApp, look promising as cost-effective tools to reduce the number of hospital face-to-face visits to deliver in a healthcare institution by the implementation of the online intervention.

#### 3.1.3. Anderson et al. (2017)

The study conducted by Anderson et al. [54] tested the usefulness of family-based treatment (FBT) delivered through a telehealth platform (i.e., a video conferencing format) for adolescents with anorexia nervosa (AN) and their families, who are unable to access this therapy in person, in order to detect any effects on AN symptoms such as weight gain, food psychopathology, mood state and self-esteem level.

Ten adolescents (13–18 years, average age 16.08) who satisfied DSM 5 diagnostic for AN or Atypical AN received 20 therapy sessions in 6 months. FBT treatment was provided according to the FBT manual for AN in adolescents in [55], which presents three phases of familial treatment of anorexia, moving from the initial phase of information and action of parents in order to take on the task of re-infecting their adolescent, to the second phase involving both adolescents and parents to negotiate a new model of relationships, up to the last phase, in which adolescent problems are examined in the context of anorexia symptoms absence. The model claims to entrust parents with the responsibility for the adolescent’s eating behaviors and gives them responsibility for the process of refeeding.

In addition to the measurement of adolescents’ BMIs, as measured at home by the parents themselves at baseline and the 6-month follow-up, the Eating Disorder Examination (EDE) [56], the Beck Depression Inventory (BDI) [57], the Rosenberg Self Esteem Scale (RSE) [58], and the Mini International Neuropsychiatric Interview for Children and Adolescents (MINI-Kid) [59] were administered. In order to assess the treatment procedures’ acceptability, Anderson et al. [54] used the following question “On a scale of 0 to 10, how useful was the treatment to you?” and obtained these results: the average score was 6.56 for adolescents, 9.11 for fathers and 9.11 for mothers. A significant improvement was detected from baseline to the end of treatment and at the follow-up of 6 months, with effect sizes from moderate to large for depression and self-esteem levels as well.

In conclusion, this study showed the effectiveness of FBT delivered exclusively via telehealth platforms (videoconferencing). Clinical results were encouraging over the study period, from baseline to end of treatment and from baseline to 6-month follow-up, on all relevant parameters, such as weight, cognition related to eating disorders, mood and self-esteem. However, limitations of this study can be seen due to its small sample size and lack of a control group with whom the results of treatment could be compared (e.g., administered in therapy as usual mode —TAU). A larger confirmatory study is needed to fully determine the effectiveness of the FBT delivery model.

#### 3.1.4. Jacobi et al. (2018)

The RCT study conducted by Jacobi et al., [60] was designed to evaluate the effectiveness of the web-based preventive program Eltern als Therapeuten (E@T) in reducing risk factors and symptoms of AN, involving adolescent parents and daughters. To be included in the study, the girls had to be between the ages of 11 and 17 and meet AN risk criteria based on screening results. The researchers defined risk as a combination of factors selected from the following three categories: (A) established risk factors for AN such as high weight and fitness problems and guidance for thinness, detected through Eating Disorder Inventory (EDI-2) subscale scores [61]; (B) early symptoms of AN indicated by low weight (defined as <90% EBW; Centers for Disease Control and Prevention, 2001) or significant weight loss (5% in the last 6 months); and (C) presence of one of the four, likely risk factors, for example, high levels of perfectionism defined by a score ≥78.0 as assessed by Frost Multidimensional Perfectionism Scale [62], amenorrhea, excessive exercise and the presence of an eating disorder in the family. To be included in the study, criterion B was mandatory, and either criterion A or C was required (or both). Parental intervention was based on the first phase of family-based treatment for Lock’s AN [55], Lock and Le Grange’s parental guide [63] and an Internet-based intervention to prevent eating disorders for adolescents [64]. The E@T intervention consisted of a web-based program of six sessions for parents accessible over the course of 6 weeks that was moderated by eating disorder experts. The intervention also included a moderated web-based discussion group for parents, weekly monitoring journals related to their daughter’s weight, nutrition and exercise with feedback provided by moderators, videos and two phone calls to allow personalized feedback on the daughter’s problems with nutrition, weight and shaping concerns.

Overall, 477 girls (447/3941, 12.10%) were identified as at risk of AN, and 256 of these were contacted. A total of 66 families (66/256, 25.8% of those contacted) were randomized to E@T (N:32) or a waitlist control condition (34). A total of 43 (43/66, 65%) participated in post-treatment assessments, and 27 (27/66, 41%) in UF at 12 months. Among the main findings [61], it was discovered that on average, parents in the intervention group opened 28% of the program pages (median 16%), participated in 2.7 out of 6 sessions and connected to the program 3.4 times. In total, 29% of randomized parents never logged into the program, and only 16% opened more than 75% of the program’s pages. A significant difference between the intervention and control groups in weight gain was observed: between pre-intervention and 12-month FU, the girls in the intervention group gained significantly more weight and faster, even if the size of the effect falls within the medium-small range. No other significant differences were found between the groups on secondary outcomes reported by children and parents. No diagnosis of DSM-IV with new-onset complete AN syndrome was observed over time in either group.

In addition, ED and weight measurements improved in participants in both groups who completed post-intervention and FU measures, which limit the possibility of seeing differences. The reasons for the improvement in the control groups were not known but may be indicative of regression to the average effects.

However, these findings must be analyzed while considering that few parents were willing to enroll and engage in the study; there was low parental involvement in the intervention and high dropout rates (i.e., more than 50% in the control group and 65.6% in the intervention group, which may have negatively affected the effectiveness of the prevention intervention).

#### 3.1.5. Yaffa et al. (2021)

The study conducted by Yaffa and colleagues [65] addressed the issue of treating eating disorders in adolescent subjects in the COVID-19 era. The need to develop this theme arises from the observation of recent surveys on adult patients with AN and bulimia nervosa (BN) [16] during the COVID-19 pandemic observing an increase in the related eating disorder (ED) symptoms, increased general anxiety and decreased quality of life. Patients reported a 39–87% increase in the frequency of food restriction, bingeing episodes and purging behaviors.

The pandemic conditions have hindered the ability to practice face-to-face clinical practice and in this context, there is limited information on the treatment of adolescent ED patients during the COVID-19 outbreak [25].

The pediatric and treatment center in Safra Children’s Hospital at Sheba Medical Center, Tel Hashomer, Israel, envisaged the implementation of interventions that included a multidisciplinary service, aimed at children and adolescents aged 6 to 18 who presented ED disorders. The treatment dedicated to patients was aimed at ensuring total care of both the individual with the symptom and their family.

The protocol used by Yaffa et al. included different types of services, including, first of all, a behavioral orientation combined with a nutritional rehabilitation program; a path of psychodynamic or individual CBT psychotherapy; individual expressive movement therapy interventions; an anorexia nervosa treatment model for adults (MANTRA) protocol [66], and group expressiveness.

From the analysis of the four cases treated in this work, it was found that the use of telemedicine has favored the process of taking charge and managing patients who presented symptoms related to ED.

In fact, by resorting to an increase in the number of sessions using multi-professional telemedicine meetings, including 37% of all sessions during the first 10 months of 2020, it was possible to guarantee, in the pandemic phase, continuous support provided by specialized professionals on the use of telemedicine, thus maintaining therapeutic continuity.

In conclusion, it can be said that the use of telemedicine treatment, while not very widespread in the pre-pandemic, has offered the possibility, even in a flexible way, of a wide range of multidisciplinary treatments, with the inclusion of the different members of the patient’s family. Additionally, for this reason a good number of subjects have chosen to continue a telemedicine path even after the resumption of face-to-face services.

#### 3.1.6. Wood S. et al. (2020)

In the study of Wood et al. (2020) [67], 392 subjects inserted in telemedicine services were involved between March 16 and April 15, 2020. The patients were followed through with in video visits for eating disorders (39%), contraception/menstrual disorders (22%), gender affirmation care (17%), general adolescent medicine (15%), HIV treatment (6%) and substance abuse (1%).

Taking care of patients followed a process of optimizing telemedicine for adolescent care. Providers interacted with patients/guardians via video via the EHR mobile application and were able to synchronously access the charts via computer for review and documentation. Initially, two providers and a patient/guardian had the opportunity to attend visits at the same time. Subsequently, the update system allowed the participation of up to five people in simultaneous visits, increasing access for interpreters, trainees, multidisciplinary team members and separated parents.

Despite the pandemic period, the service presented was a great success; in fact, looking at the data of the previous year (2019), it can be deduced that in the same period, 618 visits were completed, considering a loss of volume of 36% overall. Compared to the 2019, the no-show rate (11%) and the telemedicine no-show rate (6%) were significantly lower (p 1/4.01).

In conclusion, it can be said that geographical data reported by previous work suggests that there are also potential measured gains in health care delivery, even from telemedicine, which should be measured in future studies. Telemedicine can produce significant financial savings for families who can access care by bypassing the costs of travel, accommodation and time burden required for in-person visits away from home [68,69]. Contraceptive care was provided, avoiding higher levels of patient care through multidisciplinary prompt intervention.

## 4. Discussion

As is consistent with our initial hypotheses, it is possible to detect the effectiveness of therapeutic and prevention interventions for eating disorders in adolescents delivered through telepsychotherapy. From the analysis of the studies taken into consideration, it was found that these therapeutic interventions can produce significantly positive results after a medium- and long-term period or within a range ranging from 4 weeks to 52 weeks of observation in follow-up.

Considering the studies examined, it is possible to maintain that at present, e-health therapies for eating disorders have yet to be refined or prove to be more engaging both for those who develop forms of anorexia and for the family figures who play the role of caregiver for these patients. Despite these premises, a particular acceptance and satisfaction was observed, as well as the observation of significantly satisfactory parameters for those therapies provided in the study by Anderson et al. [54], which also involves the family of the patients, must find a way to guarantee that the latter will follow guidelines for the clinical and psychological improvement of the participants of the experimental sample.

The possibility of including treatment programs based on e-health work guarantees continuity of care and continuous monitoring by clinicians as well as a considerable decrease in the demand for treatments in outpatient services in the area [48]. Modern e-health treatments for eating disorders provide potential bases of continuous assistance and decidedly less burdensome costs of territorial services in the case that these are not identified as necessary.

The research of Heinicke et al. [42] and Anderson et al. [54] seems to be the most complete and most significant in terms of effective clinical results. The first study [42] focused its intervention on the use of an application that allows a good therapeutic alliance between patient and psychologist in the long term that promoted an increase in weight parameters and a decrease in depressive aspects.

The second study [54] focused on the direct support of not only patients but also the entire family unit, which plays a fundamental role in the process of taking charge and looking after the patient, as well as instigating and holistic improvements in the AN.

Similarly to other therapies, there are also limitations and disadvantages related to telepsychotherapy. These can differ extensively among individuals, as everybody has different inclinations for care. Hence, some disadvantages should be considered, such as extra interruptions in telepsychotherapy due to contextual noise, and the prompts in telepsychotherapy, which are more diverse than prompts in face-to-face psychotherapy since the psychologist and patient are communicating through a screen, and this may take time to get used to. Finally, some individuals may miss this absence of a face-to-face relationship.

People have positive perceptions about telepsychotherapy, and factors that may affect its impact include type of equipment and technology, education, cultural aspects, the ease-of-use of technology used, knowledge of the benefits of telehealth, privacy and costs [70,71].

Rapid telehealth conversion is achievable across a broad scope of subspecialty care for adolescents [67], eating disorders being one of the most complicated psychopathological disorders to treat in normal conditions even without the recent COVID-19 outbreak. Additionally, a recent case series used long-distance treatment (telepsychotherapy) conducted by an integrated team of dietitians, psychotherapists and psychiatrists to help the parents and patients with eating at home and showed a deterioration in the condition of the four adolescents at the start of the COVID-19 quarantine. The use of multi-professional, long-distance treatment showed an improvement in the condition of three of the four adolescents living in well-organized families with the motivation and ability to adjust to the new conditions, but the one girl who did not show improvement, whose family experienced more problems [65], emphasized once again that the role of the family is fundamental, even when the intervention is done at a distance.

## 5. Conclusions

The risks related to the exclusive use of technological tools for the care and treatment of ED could lead to high drop-out rates of subjects since the face-to-face relationship that these subjects require for the correct psychological support is not guaranteed. Despite this, it seems appropriate to focus attention also on these new opportunities for intervention, especially to avoid sudden aggravations and to create potential channels of intervention that in particular circumstances may be difficult to apply.

## Figures and Tables

**Table 1:** Legend: BMI = Body Mass Index; BSQ-SF = Body Shape Questionnaire-Short Form; BCS = Body Comparison Scale; DEBQ-R = Dutch Eating Behavior Questionnaire Restraint; EDI-B = Eating Disorder Inventory for Bulimia; SATAQ-3 = Sociocultural Attitudes Towards Appearance Scale-3; BDI-SF = Beck Depression Inventory Short Form; EDE-Q = Eating Disorder Examination Questionnaire; SEED = Short Evaluation of Eating Disorders; STAI = State-Trait Anxiety Inventory; EQ-5D-5L = EuroQoL-EQ-5L; EDE = Eating Disorder Examination; RSE = Rosenberg Self Esteem Scale; MINI-Kid = Mini International Neuropsychiatric Interview for Children and Adolescents; WCS = Weight Concerns Scale; MPS-F = Frost Multidimensional Perfectionism Scale; PMI = Parent Motivation Inventory.

Authors	Year	Title	Nation	Type of Study	Sample	Measures	Follow Up	Results
Heinicke, B. E., Paxton, S. J., McLean, S. A., and Wertheim, E. H.	2007	Internet-delivered targeted group intervention for body dissatisfaction and disordered eating in adolescent girls: a randomized controlled trial.	Australia	Randomized Controlled Trial	73	BMI BSQ-SF DEBQ-R EDI-B SATAQ BDI-SF	2 months 6 months	Significant improvements in measures of body dissatisfaction and symptoms of disordered eating (T2) in the intervention group undergoing CBT-like online sessions. These results were also maintained in the follow-up at 2 and 6 months.
Anastasiadou, D., Folkvord, F., Brugnera, A., Cañas Vinader, L., SerranoTroncoso, E., Carretero Jardí, C., Linares Bertolin, R., Muñoz Rodríguez, R., Martínez Nuñez, B., Graell Berna, M., Torralbas-Ortega, J., Torrent-Solà, L., Puntí-Vidal, J., Carrera Ferrer, M., Muñoz Domenjó, A., Diaz Marsa, M., Gunnard, K., Cusido, J., Arcal Cunillera, J., & Lupiañez-Villanueva, F	2020	An mHealth intervention for the treatment of patients with an eating disorder: A multicenter randomized controlled trial.	Spain	Randomizes controlled trial	106	EDE-Q SEED BDI-II STAI EQ-5D-5L	NO	Significant reductions in primary and secondary outcomes were observed for participants in both groups, with no differences between the experimental and control groups, demonstrating that the combined intervention between CBT and smartphone apps does not provide further improvements in symptoms.
Anderson, K. E., Byrne, C. E., Crosby, R. D., and Le Grange, D.	2017	Utilizing Telehealth to deliver family-based treatment for adolescent anorexia nervosa.	USA	Clinical trial	10	BMI EDE BDI RSE MINI-kid	6 months	The percentage of mBMI improved significantly from basaline to the end of treatment, and from baseline to FU, with medium-large effect sizes. Reduction in secondary symptoms (depression).
Jacobi, C., Hütter, K., Völker, U., Möbius, K., Richter, R., Trockel, M., Jones Bell, M., Lock, J., & Taylor, C. B.	2018	Efficacy of a Parent-Based, Indicated Prevention for Anorexia Nervosa: Randomized Controlled Trial	Germany	Randomized controlled trial	66 Families with high risk’s daughters for AN.	WCS EDI-2 MPS-F EDE PMI	6 months 12 months	The girls in the intervention group gained significantly more weight, although the size of the effect falls within the medium-small range. Parental participation in prevention intervention was very low
Yaffa S. Adi E Itai P. Marit J. Doron G. Daniel S.	2021	Treatment of eating disorders in adolescents during the COVID-19 pandemic: a case series	Israel	Case report	4 patients with ED		NO	The use of multidisciplinary interurban telemedicine treatment resulted in an improvement in the condition in three of the four adolescents living in well-organized families, only one teenager showed no improvement.
Wood M., M.S.H.P MD., White K., M.P.H MD., Peebles R., Pickel MD J., Alausa M., Mehringer J., Dowshen N., M.S.H.P. MD.	2020	Outcomes of a Rapid Adolescent Telehealth Scale-Up During the COVID-19 Pandemic	USA	Clinica Trial	392		No	In 331 unique patients, with an 82% appointment completion rate. Video visits were conducted for eating disorders (39%), contraception/menstrual disorders (22%), gender-affirming care (17%), general adolescent medicine (15%), HIV treatment (6%), and substance abuse (1%).

## Data Availability

Not applicable.
